# Nephroprotective effect of spexin in dogs and cats

**DOI:** 10.1186/s12917-026-05316-y

**Published:** 2026-02-21

**Authors:** Maciej Gogulski, Ewa Pruszyńska-Oszmałek, Natalia Leciejewska, Dawid Szczepankiewicz, Maria Nowak, Paulina Juzwik, Maciej Sassek, Jan Włodarek, Paweł Antoni Kołodziejski

**Affiliations:** 1 Department of Preclinical Sciences and Infectious Diseases, Faculty of Veterinary Medicine and Animal Science, Poznan University of Life Sciences, Wolynska 35, Poznan, 60-637 Poland; 2 University Center for Veterinary Medicine, Poznan University of Life Sciences, Szydlowska 43, Poznan, 60-656 Poland; 3 Department of Animal Physiology and Biochemistry, Faculty of Veterinary Medicine and Animal Science, Poznan University of Life Sciences, Wolynska 35, Poznan, 60-637 Poland

**Keywords:** Chronic kidney disease – CKD, Spexin, MDCK, CRKF

## Abstract

**Background:**

Kidney disease is a common and clinically significant problem in dogs and cats, yet the underlying pathophysiological mechanisms remain incompletely understood. A large part of the research concerns the role of newly described peptides/proteins that may potentially be involved in these processes. One of the peptides whose role in renal metabolism has not yet been fully understood is spexin (SPX), which has a wide distribution in the body and is involved in the regulation of many physiological processes. The aim of this study was to evaluate SPX concentration in serum from dogs and cats with chronic kidney disease (CKD) and to investigate the potential role of SPX in kidney cell metabolism using an in vitro model based on MDCK and CRFK cells.

**Results:**

Our findings showed a significant reduction in serum SPX levels in animals with CKD (*p* < 0.01). We also demonstrated, for the first time, the presence of SPX and its receptors GALR2 and GALR3 at the mRNA level in both cell lines. Moreover, we demonstrated that SPX supplementation increased cell viability in both cell lines (MDCK: *p* < 0.05; CRFK: *p* < 0.01), without affecting proliferation. Furthermore, SPX reduced the mRNA expression of key fibrotic markers associated with CKD progression, including α-SMA, TIMP1, Col1a, and fibronectin (*p* < 0.05). These effects were more pronounced following epithelial–mesenchymal transition (EMT) induction with TGF-β1 (*p* < 0.01).

**Conclusion:**

Taken together, the obtained results indicate that SPX could be a regulator of kidney cell function and may be a potential therapeutic target in the treatment of kidney diseases in dogs and cats.

**Supplementary Information:**

The online version contains supplementary material available at 10.1186/s12917-026-05316-y.

## Introduction

 Kidney disease (KD) in dogs and cats is a significant issue frequently encountered by veterinarians. It is estimated that KD affects approximately 80% of the cat population and 10% of the dog population [1]. One of the most common forms of kidney disease is chronic kidney disease (CKD), a prevalent condition in older cats and dogs leading to significant morbidity and mortality. In cats, especially those over 10 years old, CKD affects up to 30–40% of the population, making it a leading cause of health decline in senior felines [2]. In dogs, CKD is also common in older individuals, though the prevalence is slightly lower compared to cats [3]. Advancements in diagnostics and treatment have improved the quality and length of life in affected pets. Early detection and appropriate management are crucial for improving outcomes in pets with CKD [4, 5]. In addition to methods for treating and preventing these diseases in dogs and cats, it is equally important to explore the pathophysiological mechanisms associated with kidney diseases. This includes investigating the role of peptides, proteins, and hormones naturally synthesized by the body, as changes in their levels or functions may contribute to disease processes. Consequently, it is crucial to investigate the molecular mechanisms underlying CKD and to identify biologically active substances that could potentially modulate or inhibit their progression. One of them appears to be spexin (SPX). SPX is a novel and highly conserved peptide consisting of 14 amino acids, discovered in 2007 through bioinformatics approaches [6]. The biological activity of SPX is mediated via two isoforms of the galanin receptors (GALRs), GALR2 (galanin receptor isoform 2) and GALR3 (galanin receptor isoform 3). Since its discovery, numerous studies have demonstrated the beneficial effects of SPX on metabolism. Research has shown that SPX can inhibit food intake, regulate fat tissue metabolism by modulating both lipolysis and lipogenesis, and influence insulin secretion from pancreatic beta cells [7, 8]. Although the role of spexin (SPX) in model species, such as rats and mice, as well as in humans, is becoming increasingly understood, its role in the metabolism of dogs and cats remains largely unexplored. Our previous studies have demonstrated that SPX concentration in blood serum is closely correlated with body condition, as measured by the body condition score (BCS) [9].

Additionally, we have observed that SPX is expressed in peripheral tissues involved in glucose metabolism, including the adipose tissue, liver, and kidney [10, 11]. Recent studies also suggest that SPX may mitigate pathological changes associated with kidney disease, as demonstrated in laboratory rats with CKD induced by adenine hemisulfate administration [12, 13]. However, the relationship between SPX and kidney disease in other animal species has not yet been investigated.

That’s why, using blood serum obtained from cats and dogs with chronic kidney disease, we decided to investigate the potential association between CKD and serum SPX concentrations in these species. In addition, we investigated whether there is a correlation between the concentration of this peptide in blood and other indicators of kidney disease dysfunction in dogs. In the further part of the study, we decided to describe the influence of SPX on the metabolism of CRFK and MDCK cells by examining the effect of SPX on cell survival and proliferation, but also on the mRNA expression of genes related to the epithelial-mesenchymal transition, which is a common pathological consequence of CKD and leads to fibrotic processes [14].

## Materials and methods

### Animals

Blood samples from dogs and cats were collected into plastic tubes containing gel to separate serum from cellular components using routine veterinary procedures in accordance with applicable law. All procedures were performed in accordance with Polish law, as the Act on the Protection of Animals Used for Scientific or Educational Purposes was adopted in Poland on 15 January 2015. This act implements Directive 2010/63/EU of the European Parliament and the Council of 22 September 2010 on the protection of animals used for scientific purposes. Animal caregivers provided informed consent for the use of surplus blood serum, obtained during routine veterinary examinations, for research purposes. All samples were anonymized and not linked to caregiver data. The diagnostic evaluation of the animals included in the study was carried out by experienced veterinarians using standard procedures routinely applied in the diagnosis of this disorder, including blood and urine analyses, physical examination, and/or imaging techniques. The urine specific gravity in animals with renal dysfunction was below 1.035, and in dogs specifically below 1.030. In some cases, urinary sediment was present; however, in all cases included in the study, the sediment was inactive.

For the purpose of our study, the animals were divided into groups based on their serum creatinine concentrations. Dogs and cats were classified according to the following criteria: dogs — healthy group: <120 µmol/L; group 1: 130–200 µmol/L; group 2: 201–400 µmol/L; group 3: >401 µmol/L; and cats — healthy group: <150 µmol/L; group 1: 150–200 µmol/L; group 2: 201–400 µmol/L; group 3: 401–750 µmol/L; group 4: >750 µmol/L. The basic characteristics of the patients whose blood and USG were used are presented in Table 1 (cats) and Table 2 (dogs). The age and breed of animals are listed in Supplementary Tables 1 and 2. As data on BCS values for individual animals were not available, we used lipid profile data as a surrogate marker to account for the potential influence of body weight.

**Table 1:** Characteristics of experimental animals – cats

	Group 1	Group 2	Group 3	Group 4	Group 5
	Healthy	CKD1	CKD2	CKD3	CKD4
n =	9	19	23	21	12
Creatinine concentration [µmol/L]	<150	150–200	201–400	401–750	750<
Creatinine mean [µmol/L]	119.3 ± 14.23	183.1 ± 8.393	328.6 ± 46.78 (1 vs. 3; *P* = 0.002) (2 vs. 3; *P* = 0.009)	565.9 ± 96.61 (1 vs. 4; *P* < 0.001) (2 vs. 4; *P* < 0.001) (3 vs. 4; *P* < 0.001)	1242 ± 342.2 (1 vs. 5; *P* < 0.001) (2 vs. 5; *P* < 0.001) (3 vs. 5; *P* < 0.001) (4 vs. 5; *P* < 0.001)
Urine specific gravity (USG)	1.040 ± 0.008	1.028 ± 0.007 (1 vs. 2; *P* < 0.001)	1.026 ± 0.007 (1 vs. 2; *P* < 0.001)	1.025 ± 0.006 (1 vs. 2; *P* < 0.001)	1.019 ± 0.006 (2 vs. 5; *P* < 0.01) (3 vs. 5; *P* < 0.05)
Urea mean [mmol/L]	5.814 ± 2.195	13.16 ± 2.692	27.96 ± 9.859 (1 vs. 3; *P* = 0.002) (2 vs. 3; *P* = 0.01)	47.51 ± 13.89 (1 vs. 4; *P* < 0.001) (2 vs. 4; *P* < 0.001) (3 vs. 4; *P* < 0.001)	97.25 ± 30.46 (1 vs. 5; *P* < 0.001) (2 vs. 5; *P* < 0.001) (3 vs. 5; *P* < 0.001)
Triglicerides [mmol/l]	0.410 ± 0.134	0.437 ± 0.187	0.434 ± 0.238	0.430 ± 0.364	0.451 ± 0.180
Cholesterol [mmol/l]	5.404 ± 1.341	5.089 ± 1.57	5.281 ± 1.392	5.322 ± 1.615	5.314 ± 1.569
Glucose [mmol/l]	5.809 ± 1.442	5.471 ± 1.688	5.677 ± 1.496	5.696 ± 1.758	5.713 ± 1.686

Results are presented as mean ± SD

**Table 2:** Characteristics of experimental animals – dogs

	Group 1	Group 2	Group 3	Group 4
	Healthy	CKD1	CKD2	CKD3
n =	13	23	28	13
Creatinine concentration [µmol/L]	< 120	130–200	201–400	401<
Creatinine mean [µmol/L]	104.4 ± 12.16	158.7 ± 18.65	271.4 ± 63.87 (1 vs. 3; *P* < 0.001) (2 vs. 3; *P* = 0.003)	588.9 ± 256.6 (1 vs. 4; *P* < 0.001) (2 vs. 4; *P* < 0.001) (3 vs. 4; *P* < 0.001)
Urea mean [mmol/L]	6.365 ± 1.381	21.76 ± 11.58 (1 vs. 2; *P* < 0.02)	28.63 ± 14.24 (1 vs. 3; *P* < 0.001)	60.59 ± 25.35 (1 vs. 4; *P* < 0.001) (2 vs. 4; *P* < 0.001) (3 vs. 4; *P* < 0.001)
Urine specific gravity (USG)	1.034 ± 0.009	1.022 ± 0.007 (1 vs. 2; *P* < 0.001)	1.019 ± 0.008 (1 vs. 3; *P* < 0.001)	1.015 ± 0.007 (1 vs. 4; *P* < 0.001)
Triglicerides [mmol/l]	0.670 ± 0.331	0.662 ± 0.354	0.653 ± 0.289	0.680 ± 0.254
Cholesterol [mg/dl]	5.138 ± 1.553	4.89 ± 1.779	5.098 ± 1.366	5.398 ± 1.236
Glucose [mmol/l]	4.737 ± 0.646	4.154 ± 1.184	4.189 ± 0.864	4.579 ± 1.40

Results are presented as mean ± SD

### Cells and reagents

In vitro research was conducted on CRFK (BVD Ag-negative) and MDCK cell lines, obtained from the European Collection of Authenticated Cell Cultures (ECACC, England) and the American Type Culture Collection (ATCC, USA), respectively. Novazym Sp synthesized feline/Canine SPX. z o.o. (Poland). Eagle’s Minimum Essential Medium (EMEM) and Fetal Bovine Serum (FBS) were acquired from Corning (Tewksbury, Massachusetts, USA) and/or from Capricorn Scientific (Ebsdorfergrund, Germany). Unless otherwise specified, all other reagents were sourced from Merck/Sigma Aldrich (USA).

### Cell culture

The MDCK and CRFK cells were cultured in EMEM supplemented with 10% FBS, 100 U/mL penicillin, and 100 µg/mL streptomycin, at 37 °C in a humidified atmosphere containing 5% CO₂ according to manufacturer instructions. The medium was changed every 2–3 days, and cells were subcultured at approximately 80–90% confluence using 0.25% trypsin-EDTA. Experiments were performed using experimental medium (FBS was replaced by the addition of 0.2% bovine serum albumin - BSA).

### MTT. Neutral red uptake (NRU)

The MTT ((3-[4,5-dimethylthiazol-2-yl]-2,5 diphenyl tetrazolium bromide) assay was used to evaluate cell viability based on mitochondrial function. Cells were seeded in a 96-well plate and incubated for 24 h under standard culture conditions (37 °C, 5% CO₂). Following the incubation period, the cells were exposed to SPX in concentrations of 1, 10, 100, and 1000 nM for 24 h in experimental medium. After treatment, 20 µL of MTT solution (5 mg/mL in PBS) was added to each well, and the plate was incubated for 3–4 h at 37 °C to allow mitochondrial dehydrogenases to reduce the MTT into formazan crystals. The resulting formazan was solubilized by adding 100 µL of DMSO and incubating for 25 min at room temperature. The absorbance was measured at 570 nm using a Synergy H1 microplate reader (Biotek, USA). Cell viability was expressed as a percentage relative to the untreated control group.

The Neutral Red Uptake (NRU) assay was performed to assess lysosomal integrity and cell viability. Cells were prepared and incubated as described above. After exposure to SPX, the medium in the cells was replaced with 100 µL of Neutral Red solution (50 µg/mL in culture medium) added to each well. Cells were incubated for an additional 2–3 h at 37 °C to allow NR uptake into viable cells. After incubation, the dye-containing medium was removed, and the cells were gently washed with PBS. The internalized dye was extracted using a solution of 50% ethanol and 1% acetic acid and incubated for 10–15 min with gentle shaking. The absorbance was measured at 540 nM.

### BrDu assay

Cell Proliferation ELISA BrdU was used to determine the SPX effect on MDCK and CRFK cell proliferation (Roche, Germany). All procedures were performed according to the manufacturer’s instructions as we previously described [10].

### RNA and cDNA synthesis

RNA was isolated from the biological material using EXTRAZOL (DNA, Gdansk, Poland). cDNA synthesis was performed using 1 µg of total RNA, High-Capacity cDNA Reverse Transcription Kit (cat. no. 4374967, Applied Biosystem, USA) according to the manufacturer’s instructions.

### Real time PCR

Quantitative Real-time PCR (qPCR) was performed using gene-specific primers (see Table) and 5× HOT FIREPol^®^ EvaGreen^®^ qPCR Mix Plus (ROX) on a QuantStudio™ 12 K Flex Real-Time PCR System (Life Technologies, Grand Island, NY, USA). The specificity of the amplified products was verified by melting curve analysis with a ramp rate of 0.1 °C/s. Relative gene expression levels were calculated using the 2^–ΔΔCT method with *GAPDH* serving as the reference gene. Primers used for the reaction are placed in Supplementary Table 3.

### SPX determination in serum blood

Serum concentration of SPX in dogs and cats was determined as we previously described [10]. Using a commercially available Human Spexin Elisa Kit (Cat. no. 201-12-7257, Biotechnology Company, Shanghai, China; Intra-Assay: CV < 10%. Inter-Assay: CV < 12%). We determined the concentration of SPX in blood serum. Because the amino acid sequence of SPX is identical in dogs and cats, no additional test validation was required. However, for verification, we analyzed two dilutions (1:2 and 1:4) of three randomly selected samples from both species, and the measured values corresponded appropriately to the dilutions.

### Statistical analysis

Statistical analyses were performed using GraphPad Prism 10 (GraphPad Software, USA). Results are presented as the arithmetic mean ± standard deviation (SD). Statistical analyses were performed using a one-way ANOVA test with a Dunnett post-hoc test compared to the control group. The normality of distribution in each group was assessed using the D’Agostino & Pearson test. Statistical significance was defined as *p* < 0.05 (*) and *p* < 0.01 (**). Relationships between spexin concentration and creatinine and urea in blood were analyzed using Pearson’s correlation model and linear regression.

## Results

### Spexin serum concentration

**Fig. 1:**
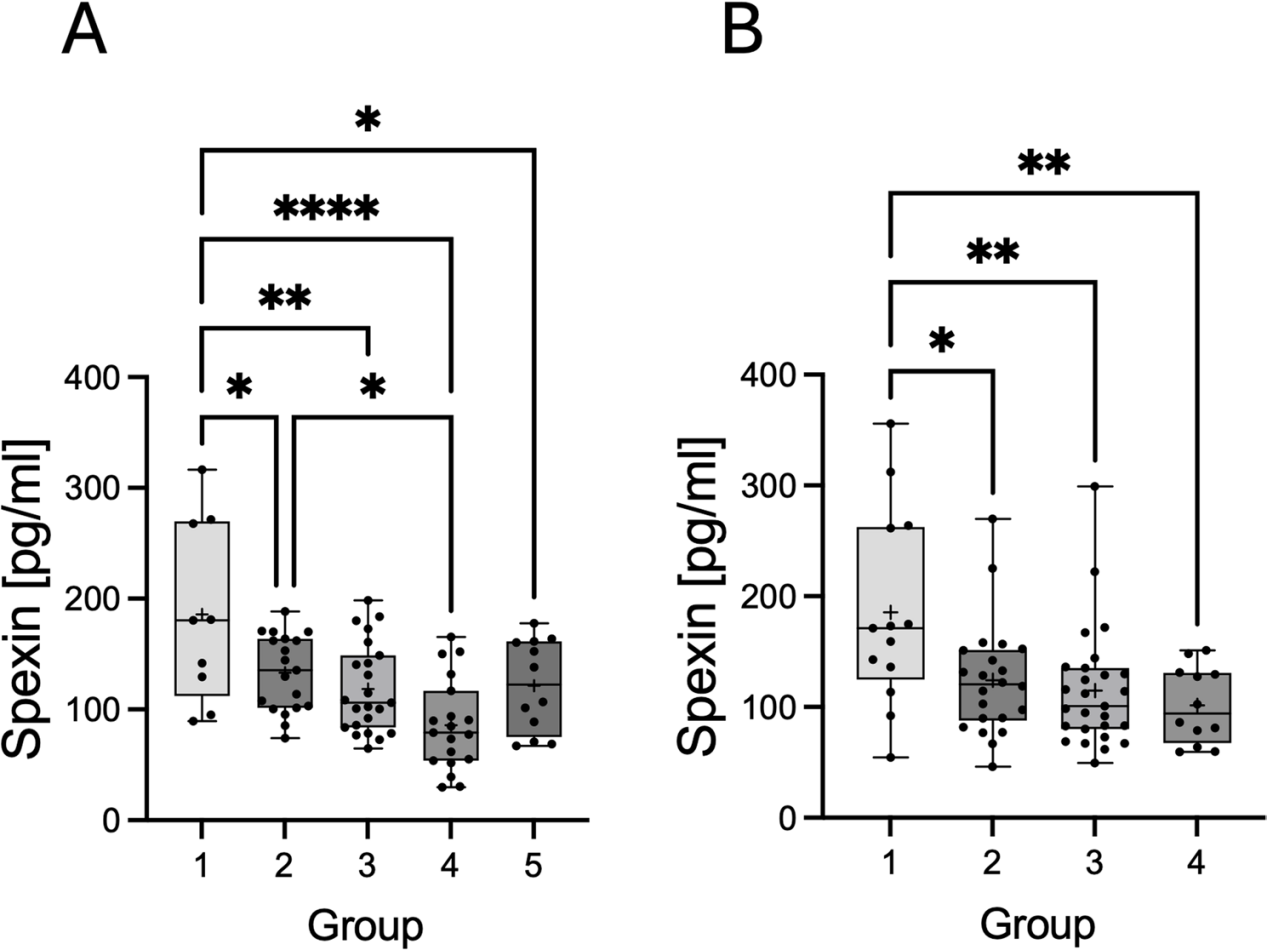
Serum SPX concentration in cats (**A**) and dogs (**B**) with CKD. The boxes represent the 25th and 75th quartiles, with the horizontal line representing the median. Moreover, the graph presents individual values as dots and the arithmetic mean as “+”. The whiskers represent the range of the data. Statistically significant differences between the experimental groups are marked as: *P < 0.05; **P < 0.01; ****P < 0.0001

A statistically significant decrease in SPX concentration was demonstrated in the blood serum of dogs and cats with CKD. In the case of cats, the concentration was lower in all groups compared to the control group (1 vs. 2, *P* < 0.05; 1 vs. 3, *P* < 0.01; 1 vs. 4, *P* < 0.001; 1 vs. 5, *P* < 0.05). Moreover, we also observed differences between groups 2 and 4 (*P* < 0.05) (Fig. 1A). In the case of dog serum, a difference was shown between the control group, healthy dogs (1 vs. 2, *P* < 0.05; 1 vs. 3, *P* < 0.01; 1 vs. 4, *P* < 0.01) (Fig. 1B). Moreover, using Pearson’s correlation model and linear regression, we observe negative correlation between serum concentration of SPX and creatinine in cats (Supplementary Fig. 1B; *r*=-0.3385; *P* < 0.01) and dogs (Supplementary Fig. 1D; *r*=-0.2318; *P* < 0.05). We didn’t observe a correlation between SPX and Urea in cats and dogs (Supplementary Fig. 1A and C).

### mRNA expression of SPX, GALR1, GALR2 and GALR3

**Fig. 2:**
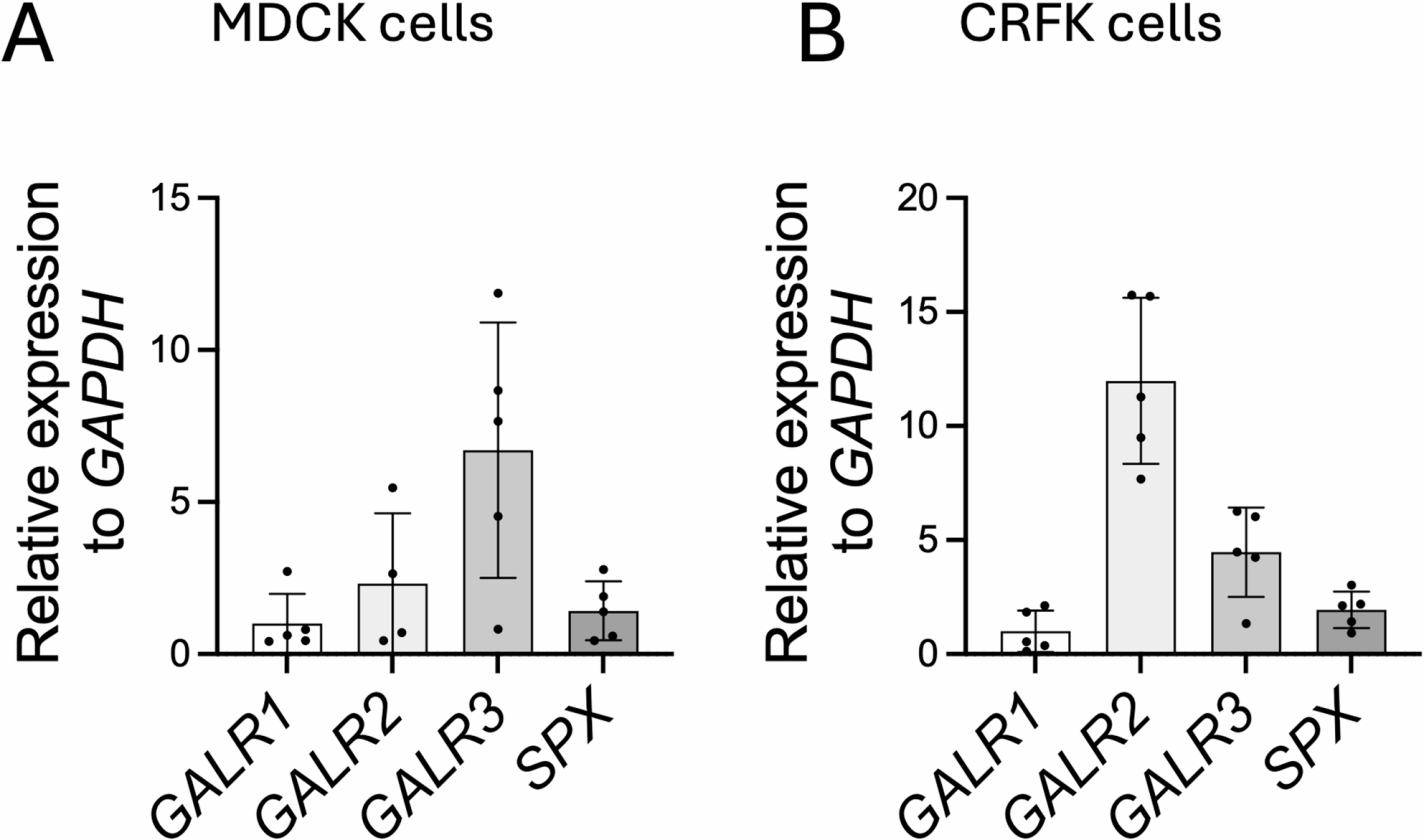
Expression of GALR2 and GALR3 mRNA in MDCK (**A**) and CRFK (**B**) cells

In the first stage of our in vitro study, we decided to check whether both SPX and galanin receptors (1,2,3) are expressed in these cells. We are aware that only isoforms 2 and 3 are indicated as SPX receptors in the literature, but we chose to check the presence of all of them. We demonstrated that all the genes studied are present at the mRNA level in MDCK and CRFK cells, though the expression profiles were different. In the case of MDCK cells, we observed relatively higher expression of the GALR3 receptor (Fig. 2A), while in CRFK cells, the highest expression was shown by GALR2 (Fig. 2B).

### Proliferation and cell viability

**Fig. 3:**
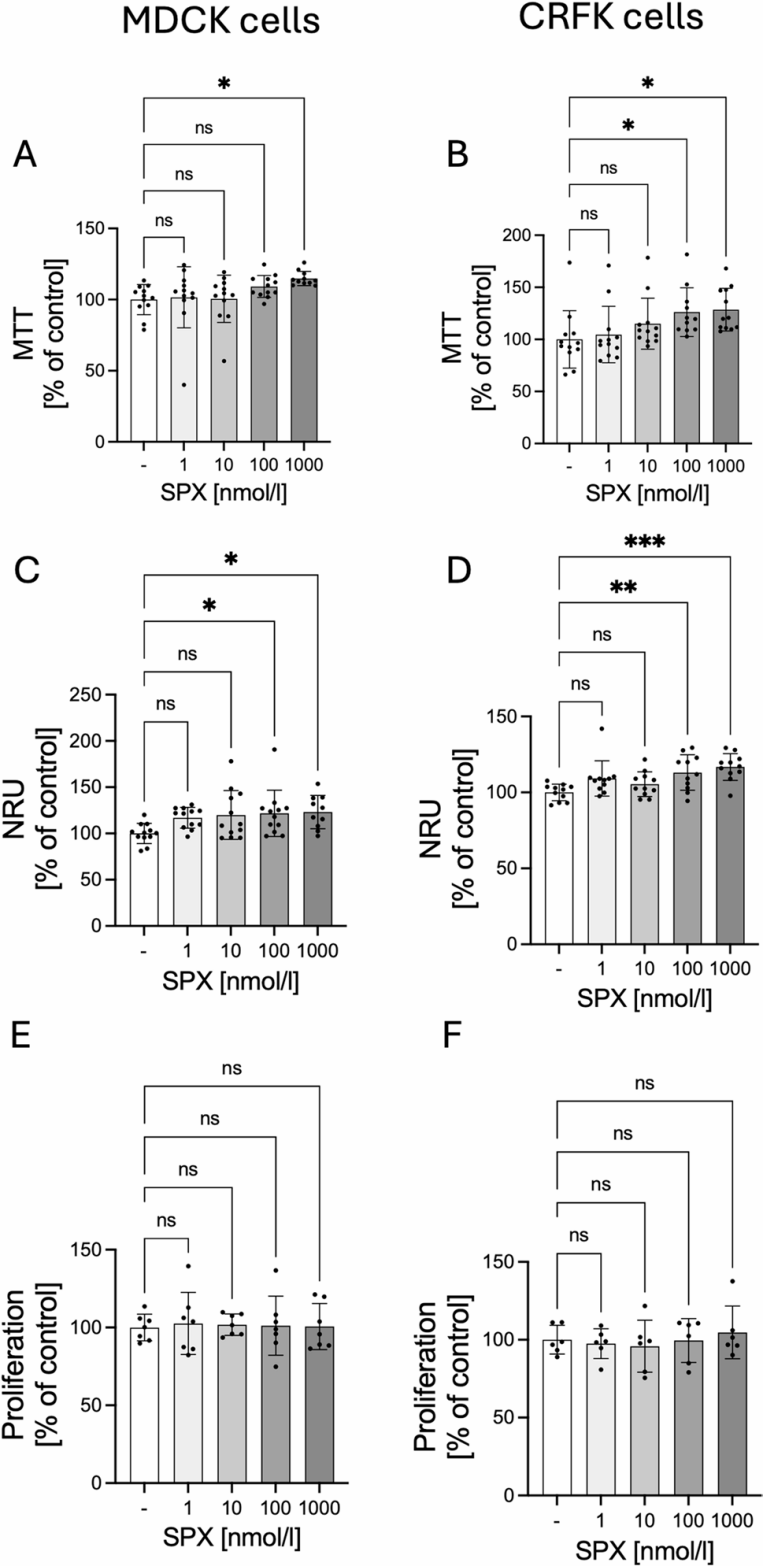
Effect of SPX on the cell viability using MTT method (**A**, **B**), using NRU method (**C**, **D**), and proliferation (**E**, **F**) in MDCK (left panel) and CRFK cells (right panel). Results are presented as mean ± SD (MTT and NRU: n = min. 5; BrDU *n* = 3). All experiments were duplicated. Statistically significant differences were determined using One-Way ANOVA with a Dunnett post-hoc test compared to the control group (without SPX). Statistically significant differences were marked as: *P < 0.05; **P < 0.01; ***P < 0.001

The next stage of the experiment was to examine the effect of SPX on the survival of MDCK and CRFK cells using two commonly used colorimetric tests, MTT and NRU. A statistically significant effect of SPX on increasing the survival of both MDCK and CRFK cells was demonstrated. In the case of the MTT test, the addition of 1000 nmol/l SPX increased the survival of MDCK cells (Fig. 3A: *P* < 0.05), while an increase in the survival of CRFK cells was demonstrated with the addition of 100 and 1000 nmol/l (Fig. 3B: *P* < 0.05). An increase in survival was also observed using the NRU test. In MDCK cells, a statistically significant increase was demonstrated with the addition of 100 and 1000 nmol/l (Fig. 3C: *P* < 0.05). A similar effect was observed in CRFK cells (Fig. 3D: 100 nmol/l, *P* < 0.01; 1000 nmol/l, *P* < 0.001). Using the BrDu method, we didn’t note any effect of SPX on the proliferation of MDCK (Fig. 3E) and CRFK (Fig. 3F) cells.

### Effect of SPX on mRNA expression of α SMA (alpha smooth muscle actin), TIMP1 (tissue inhibitor of matrix metalloproteinase 1), Col1a (collagen type I alpha 1 chain), and fibronectin (basal and stimulated by TGF-β1)

**Fig. 4:**
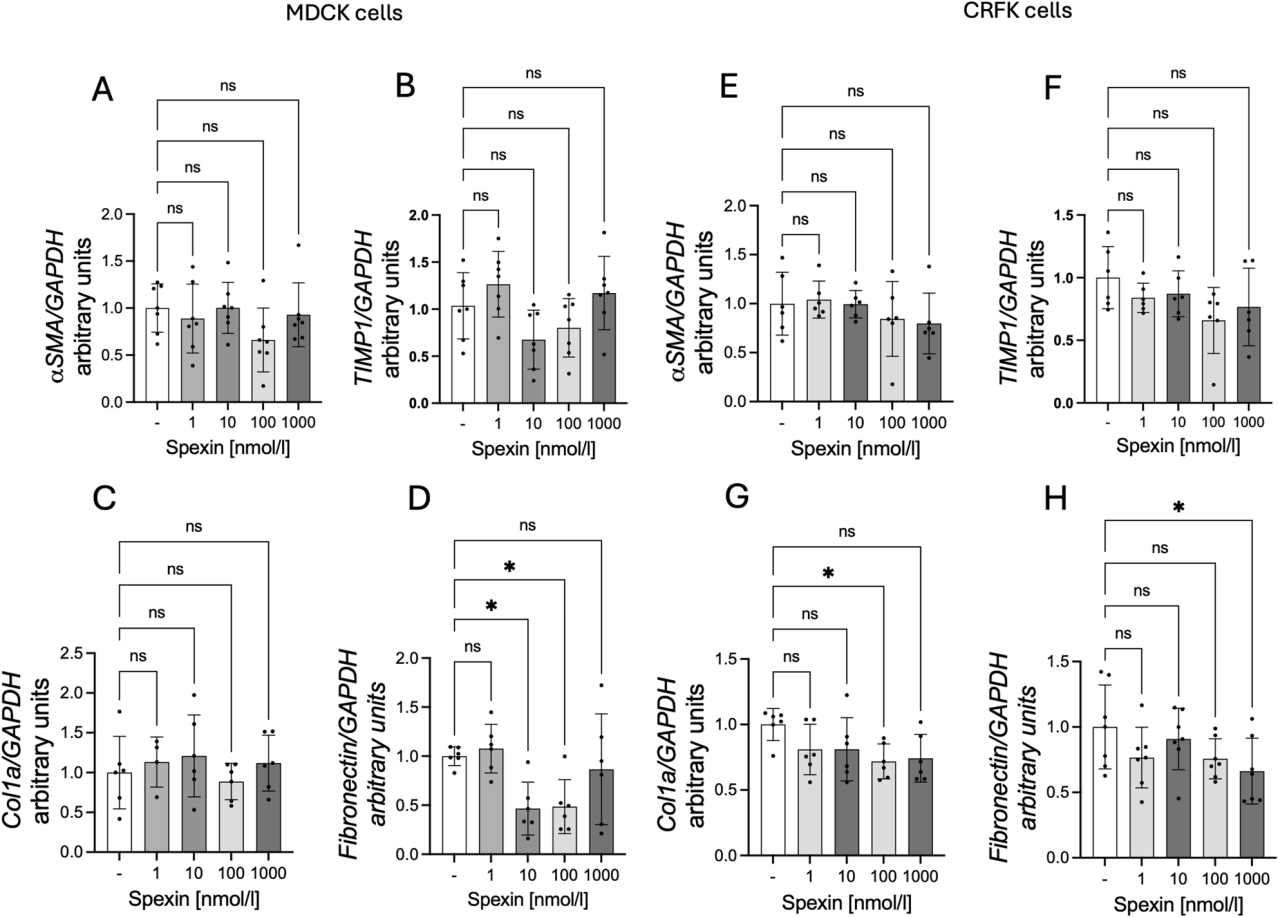
Effect of SPX on *α-SMA* (**A**, **E**), *TIMP1* (**B**, **F**), *Col1a* (**C**, **G**) and fibronectin (**D**, **H**) mRNA expression in MDCK cells (left panel) and CRFK cells (right panel). Results are presented as mean ± SD (n = min. 3). All experiments were duplicated. Statistically significant differences were determined using One-Way ANOVA with a Dunnett post-hoc test compared to the control group (without SPX). Statistically significant differences were marked as: *P < 0.05;

As mentioned in the introduction, the consequence of CKD is epithelial-mesenchymal transformation of cells. Therefore, we decided to check whether SPX affects this process. Using markers of this process, such as *α*-SMA, TIMP1, Col1a, and fibronectin. We observed decreased mRNA levels of genes encoding fibronectin with the addition of 10 and 100 nmol/l SPX in MDCK cells (Fig. 4D: *P* < 0.05). In the case of CRFK cells, we observed a statistically significant decrease in the expression of the Col1a gene (Fig. 4G: 100 nmol/l; *P* < 0.05) and fibronectin with the addition of 1000 nmol/l (Fig. 4H: *P* < 0.05).

**Fig. 5:**
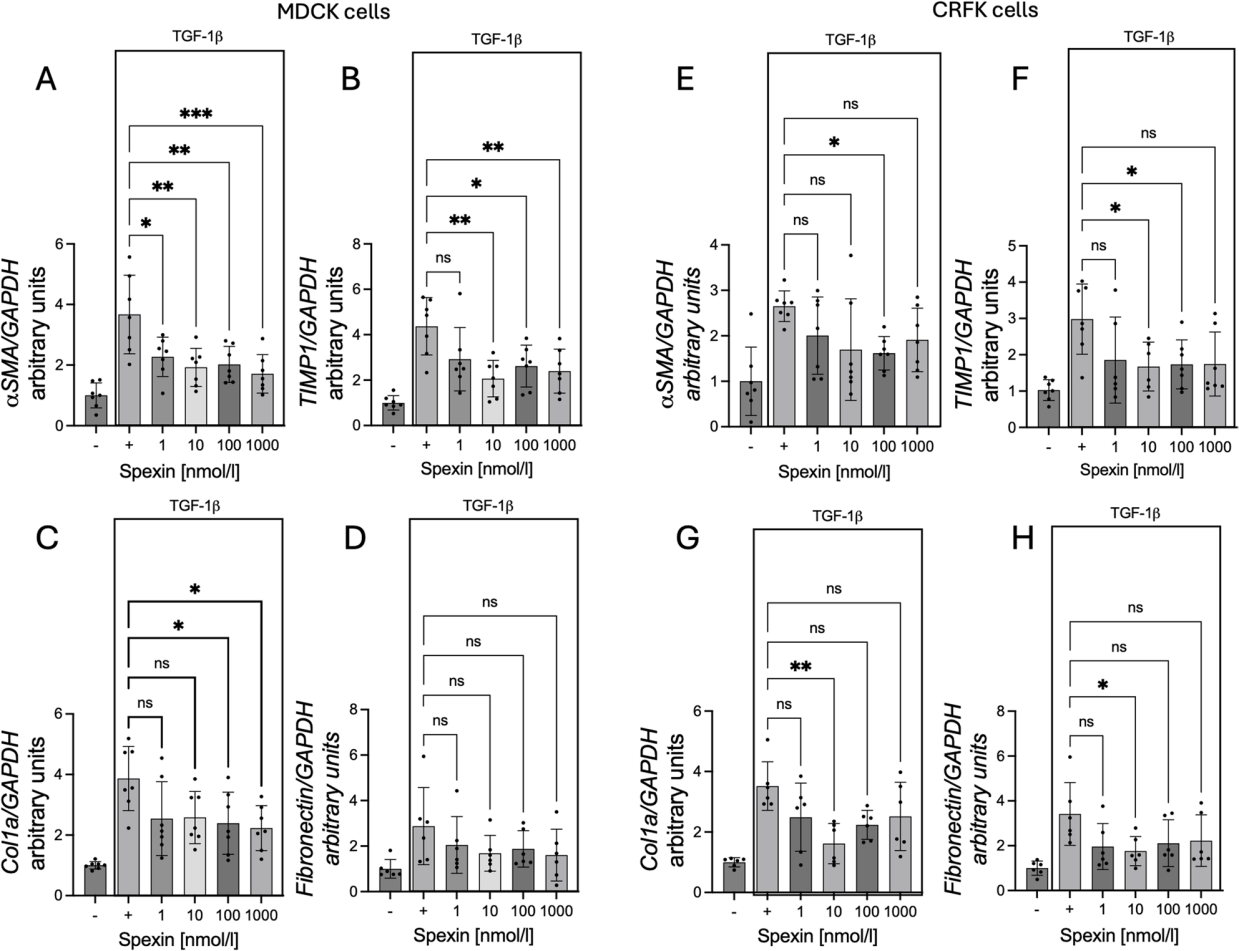
Effect of SPX on *α-SMA* (**A**, **E**), *TIMP1* (**B**, **F**), *Col1a* (**C**, **G**) and fibronectin (**D**, **H**) mRNA expression in MDCK cells (left panel) and CRFK cells (right panel) treated with TGF-β1 to induce EMT. Results are presented as mean ± SD (n = min. 3). All experiments were duplicated. Statistically significant differences were determined using One-Way ANOVA with a Dunnett post-hoc test compared to the control group (group treated only with TGF-β1). Statistically significant differences were marked as: *P < 0.05; *P < 0.05; **P < 0.01; ***P < 0.001

The final stage of the study aimed to investigate the effect of spexin (SPX) on the expression of marker genes involved in the epithelial-mesenchymal transition (EMT) process induced by TGF-β1. For this purpose, cells were treated with TGF-β1 at a concentration of 10 ng/ml, both with and without SPX. The results showed that SPX significantly reduced the mRNA levels of EMT-related genes in both MDCK and CRFK cell lines. In MDCK cells, a notable decrease in *α-SMA* gene expression was observed at all tested SPX concentrations: 1 nmol/l (*P* < 0.05), 10 and 100 nmol/l (*P* < 0.01), and 1000 nmol/l (*P* < 0.001) (Fig. 5A). A similar trend was noted for *TIMP1* gene expression, which significantly changed following SPX treatment at 10 and 1000 nmol/l (*P* < 0.01) and at 100 nmol/l (*P* < 0.05) (Fig. 5B). Furthermore, Col1a gene expression was significantly reduced at SPX concentrations of 100 and 1000 nmol/l (Fig. 5C: *P* < 0.05). In contrast, no statistically significant changes were found in the mRNA levels of the gene encoding fibronectin. In CRFK cells, a decrease in the expression of the following genes was observed: *α-SMA* after 100 nmol/l SPX treatment (Fig. 5E: *P* < 0.05), *TIMP1* after 10 and 100 nmol/l SPX treatment (Fig. 5E: *P* < 0.05), Col1a (Fig. 5G: *P* < 0.01) and fibronectin after 10 nmol/l SPX treatment (Fig. 5H: *P* < 0.05).

## Discussion

In the present study, we report for the first time serum spexin is downregulated in cats and dogs with increased serum urea and creatinine concentration. We also showed for the first time that mRNA of SPX, GALR1, 2, and 3 is expressed in MDCK and CRFK cells. Using an in vitro method, we demonstrated that SPX regulates cell viability but does not influence proliferation; however, SPX can regulate the mRNA expression of genes involved in EMT in CRFK and MDCK cells. Our findings indicate that SPX downregulates α-SMA, TIMP1, Col1a, and fibronectin, with the effect being more pronounced after TGF-β1 stimulation of the EMT process.

To investigate whether spexin (SPX) might be involved in the pathophysiology of kidney disease, we assessed its serum concentration in dogs and cats diagnosed with CKD, characterized by elevated urea and creatinine levels. We observed a reduction in serum SPX concentration in animals with CKD; however, this decrease directly correlated with the concentrations of creatinine but not a urea. Currently, the function of SPX in renal dysfunction has not been widely discussed in the literature. Most research to date on SPX has focused on its role in the regulation of carbohydrate and lipid metabolism, particularly in the context of metabolic disorders such as obesity and diabetes [15–17]. To date, the number of data on changes in serum SPX concentration in renal dysfunction is limited. In 2024, Liu et al. demonstrated that SPX levels are reduced in patients with diabetic peripheral neuropathy (DPN). Moreover, they also noted that SPX levels varied depending on the severity of pain symptoms associated with this condition [18]. This confirms previous reports that peptides derived from the prohormone proNPQ/spexin are potent central modulators of renal function [19]. This is also confirmed by our results obtained on dogs and cats. On the other hand, a study by Kay et al. (2022) demonstrated increased SPX expression in rat kidneys and elevated serum SPX levels following kidney damage induced by aluminum exposure. The authors proposed two potential interpretations: the upregulation of SPX may represent a compensatory response to tissue injury acting as a protective mechanism against metabolic stress or, alternatively, SPX may be involved in the development of pathological changes. This latter hypothesis is supported by the finding that treatment with N-acetylcysteine (NAC) reduced both SPX expression and serum concentration, which was accompanied by decreased oxidative stress markers [20]. Hence, there is a need for further research, as changes in SPX concentration may depend on the type of disease.

The first studies suggesting a beneficial effect of SPX on kidney metabolism were published in 2021 by Yazgan et al., Memi & Yazgan, and El Saka et al. [12, 13, 21]. They showed that the effect of SPX is achieved by influencing the modulation of inflammation. However, it should be noted that existing studies regarding the effect of SPX on kidney functions are based almost exclusively on in vivo models involving SPX administration to laboratory animals. Although in vivo studies are considered superior and more reliable, they have certain limitations, primarily the inability to eliminate confounding systemic factors that may influence the action of the peptide. Considering these results, it seems important to undertake studies using various techniques, both in vivo and in vitro, to describe the role of SPX in the development of kidney disorders. Our results as well as literature data prompted us to attempt to explore this issue based on in vitro models. In our study, similar to previous research, we used SPX concentrations ranging from 1 to 1000 nM. The application of these doses allowed us to determine whether the observed effects were associated with physiological or pharmacological concentrations, the latter exceeding levels typically found in circulating blood. However, it should also be emphasized that, in addition to circulating SPX that reaches the kidneys through the bloodstream, local renal production of SPX has been demonstrated. This justifies the use of higher concentrations in in vitro experiments. Evidence for local SPX synthesis comes from studies showing its expression both at the mRNA and protein levels within renal tissues [22, 23] .

In the initial stage of our in vitro experiments, we aimed to determine whether SPX and its receptors, GALR2 and GALR3, are expressed in MDCK and CRFK cells. This step was essential for the continuation of our study, as the absence of at least one of these receptor isoforms would preclude the potential activity of SPX. Our results confirmed the presence of both GALR2 and GALR3 in the tested cell lines, suggesting that SPX may indeed participate in the regulation of renal cell function. These findings are consistent with previous reports on the expression of galanin receptors in the kidneys of other species, such as rats and humans (including HEK293 cells), where both GALR2 and GALR3 isoforms have been detected. Our results, therefore, support and extend these earlier observations, confirming the presence of SPX signaling components in canine and feline kidney-derived cells [24–26]. Moreover, previous studies in mice have demonstrated that a spexin-based GALR2 receptor agonist can alleviate renal injury in a model of type 2 diabetes, suggesting a potential pathway through which SPX may exert its effects in the kidneys. Another metabolic pathway potentially regulated by the GALR2 receptor and considered relevant to the development of chronic kidney disease (CKD), is the activation of large-conductance calcium-activated potassium (BK) channels. Research by Pan et al. (2014) indicates that GALR2 receptor activation stimulates BK channel activity via the IP₃ signaling pathway, a mechanism increasingly recognized for its role in CKD pathophysiology. Calcium-activated potassium channel (BK) is widely expressed in kidneys, but in the occurrence of CKD its expression is altered, which is also accompanied by increased expression of fibrotic markers such as fibronectin, α-SMA, Col1a [27]. Furthermore, as studies have shown, BK deficiency can increase the expression of TGF-β1, which is a potent stimulator of fibrosis [28]. Therefore, it is indicated that BK activation exerts antifibrotic effects on renal fibrosis by inhibiting the TGF-β signaling pathway via accelerating TGF-β receptor degradation [26]. It should be noted that the proposed mechanism involving GALR2 and BK channels is interesting but highly speculative based on current data. However, the observed results and the causal chain described in the literature prompted us to undertake further research on the effects of SPX on proliferation, viability, and fibrosis [29–32].

The effect of spexin (SPX) on cell survival and proliferation varies by cell type, showing stimulatory actions in pancreatic β-cells and muscle cells [8, 29, 31, 33] but no effect in 3T3-L1 adipocytes [10]. On the other hand, we can find studies that indicate that SPX limits adrenocortical and granulosa cell proliferation [30]. Results obtained on CRFK and MCDK showed that SPX treatment appears to increase cell survival, but the effect on their proliferation is not visible. This clearly indicates a limitation of apoptosis processes by SPX. These results are consistent with those previously obtained in vivo in a rat model by El-Saka et al. (2021), who studied the effect of SPX on renal dysfunction in experimentally obese rats and showed that SPX administration caused a change in the expression of caspase 3 at the protein level in the kidneys of animals receiving this peptide, which also confirms the antiapoptotic effect of SPX in the kidneys [21]. Similar results were obtained using spexin-based GALR2 agonist (NS200), the administration of which to mice resulted in a reduction of the glomerulosclerosis index (GSI), which also indirectly indicates the antiapoptotic effect of SPX in the kidneys [34]. Based on the obtained results concerning the role of SPX in the survival of CRFK and MDCK cells in comparison with the literature data, it should be stated that SPX increases cell survival by limiting apoptotic processes in the kidneys.

The next process we studied was the effect of SPX on the expression of EMT markers, which is one of the pathological consequences of CKD. The results of our studies on changes in mRNA expression of the studied genes showed that SPX reduced the expression of fibronectin in MDCK cells and, Col1a, fibronectin in CRKF cells in the basal system. The mild effect of SPX on the decrease of these markers in the normal physiological state, without stimulation of this process, was observed; however, after stimulation of this process with TGF-1b in the presence or absence of SPX, we stated a significant effect of different doses of SPX on the decrease of mRNA expression of α-SMA, TIMP1, Col1a, and fibronectin. Previous literature data also indicated a possible participation of SPX in the regulation of the expression of these genes in the kidneys, as well as in other tissues susceptible to fibrotic processes, such as the liver. An example of such studies is the results of Abulfadle et al. (2022), who showed that SPX administration to rats with polycystic ovary syndrome (PCOS) caused a decrease in the expression of α-SMA and apoptotic markers in the liver [35]. In addition, some studies have shown that SPX has a protective effect on renal tissue by reducing tubular dilatation, inflammatory infiltration, necrosis, apoptosis, and fibrosis in rats on a high-fat diet [21]. Similarly, SPX treatment reduced the levels of tissue inhibitor of metalloproteinase 1 (TIMP-1) and kidney injury molecule-1 (KIM-1) in the adenine-induced chronic renal failure (CRF) rat model. Furthermore, SPX treatment reduced the levels of matrix metalloproteinase-2 (MMP2), which regulates the fibrosis process. These results, as well as our findings, indicate a protective effect of SPX on kidney injury by regulation of inflammation as well as fibrosis processes [12, 36].

We are also aware of the limitations of our study. One of the primary constraints is that the serum samples from dogs and cats used to assess spexin (SPX) concentrations were obtained during routine veterinary procedures. The data were anonymized, and only limited background information, such as breed and age, was available. Since SPX levels are known to correlate with adiposity, it would have been beneficial to assess the animals’ lipid profiles, which could serve as indirect indicators of potential lipid metabolism disorders. This would have helped to control for confounding variables and improve the reliability of the findings. However, to limit this effect, we selected animals for the study with similar glucose, triglyceride, and cholesterol concentrations. Another limitation lies in the use of ELISA kits that were not species-specific. Although the assays were validated and used previously in our studies on SPX and obesity, the measured concentrations were significantly higher than those obtained with radioimmunoassay (RIA) methods [9]. This discrepancy may result from various factors, but is most likely due to the antibody binding efficiency of canine and feline SPX, caused by a single amino acid difference in the SPX peptide sequence compared to that of humans, mice, or rats, despite the high degree of sequence conservation. Such variability is frequently observed with research-grade assays, which are not optimized for diagnostic precision. Similar inconsistencies have also been reported in other species, where SPX concentrations measured within the same species may vary by several orders of magnitude, even among healthy individuals [18, 37–40]. The most accurate method for determining the absolute concentration of circulating SPX would involve chromatographic techniques. Such analyses would need to be performed and directly compared with the results obtained using commercially available immunoassay kits in order to validate their accuracy. We are also aware of the limitations arising from the study group being characterized using only two main parameters (creatinine and urea), without including data consistent with the IRIS classification, such as urine specific gravity, urine protein-to-creatinine ratio, hematocrit or PCV, serum phosphate concentration, and blood pressure. This limitation stems from the fact that we used leftover serum samples obtained from routine veterinary testing. Nevertheless, disease classification was performed by an experienced clinician. Another limitation of our study may be the fact that some uremic animals might have had concurrent pancreatitis, which could have contributed to elevated biochemical values. In addition, due to reduced glomerular filtration rate (GFR), impaired cholesterol excretion may also occur. Unfortunately, we were not able to include these aspects in our dataset and therefore cannot exclude their possible influence on the obtained results [41, 42]. Importantly, these are the first studies of their kind, providing initial insight into the potential role of SPX, which warrants further investigation. We also acknowledge that the research models employed are not optimal for this type of study, particularly the use of CRFK cells. Some reports suggest that these cells display a phenotype more similar to fibroblasts than to kidney epithelial cells [43]. CRFK cells, which exhibit a fibroblast-like phenotype, represent a significant limitation in a study focused on epithelial–mesenchymal transition (EMT). Our rationale for including CRFK cells alongside MDCK renal epithelial cells was based on the fact that CRFK cells are of renal origin and are widely used in veterinary nephrology research, particularly in studies on renal inflammation, viral infection, and fibrosis-related mechanisms in cats. Despite their fibroblast-like morphology, CRFK cells retain the expression of several renal markers. They are known to respond to profibrotic and proinflammatory stimuli in a manner that reflects processes observed in diseased feline kidneys. Due to this fact, findings using CRFK cells primarily inform on fibroblast activation and mesenchymal signaling, which may occur in advanced stages of renal fibrosis, rather than representing a true epithelial-to-mesenchymal transition [44]. Nevertheless, due to the limited availability of alternative models, CRFK cells are commonly used as a surrogate in this type of research [3, 45].

Another limitation of our study is that the effect of SPX on the epithelial-mesenchymal transition (EMT) was assessed exclusively at the mRNA level. While gene expression analysis provides valuable preliminary insights, it does not fully reflect protein-level changes or functional outcomes. In future studies, we plan to investigate the metabolic pathways through which SPX acts on kidney cells, with a particular focus on downstream signaling mechanisms and protein expression.

In conclusion, we have demonstrated for the first time that SPX concentration changes during the occurrence of CKD in dogs and cats and that SPX can regulate cell survival processes and downregulate the expression of mRNA genes involved in fibrotic processes in MDCK and CRFK cells. It is important to emphasize that this research represents the first attempt to explore the potential role of SPX in kidney disorders in dogs and cats. Nevertheless, we acknowledge that further studies are essential to fully elucidate its biological functions and to evaluate possible therapeutic implications.

## Supplementary Information

Supplementary Material 1

## Data Availability

The datasets used and analysed during the current study are available from the corresponding author on reasonable request.

## Abbreviations

BCS: Body condition score
BrDu: 5-Bromo-2’-deoxyuridine
BSA: Bovine serum Albumin
CKD: Chronic kidney disease
Col1a: Collagen Type I Alpha 1 Chain
EMT: Epithelial–mesenchymal transition
FBS: Fetal bovine serum
GALR1: Galanin receptor 1
GALR2: Galanin receptor 2
GALR3: Galanin receptor 3
GALRs: Galanin receptors
MTT: (3-[4,5-dimethylthiazol-2-yl]-2,5 diphenyl tetrazolium bromide
NRU: Neutral Red Uptake
SPX: Spexin
TGF-β1: Transforming Growth Factor-beta 1
TIMP1: (Tissue Inhibitor of Matrix Metalloproteinase 1
αSMA: Alpha smooth muscle actin
