# Supplementary material for: Nephroprotective effect of spexin in dogs and cats
Source: BMC Vet Res. 2026 Feb 21;22:194. doi: 10.1186/s12917-026-05316-y (PMC13032655; doi:10.1186/s12917-026-05316-y)
Supplement: Supplementary file 1 — Supplementary Material 1 [file 12917_2026_5316_MOESM1_ESM.pdf]

Supplementary table 1: Characteristics of cats

Sex	Age	Breed	Clasification (group)
♂	13 years 5 months	European	1
♀n	7 years 2 months	Mix	1
♂	16 years	Mix	1
♂	11 years 11 months	Mix	1
♂	9 years	Persian	1
♂	15 years	European Shorthair	1
♂	9years	European Shorthair	1
♂	4 years 9 months	Maine Coon	1
♂	13 years 8 months	European Shorthair	1
♂	9 years 8 months	Persian	1
♂	19 years 8 months	European Shorthair	1
♂	6 years 1 month	Mix	1
♀	15 years 8 months	European Shorthair	1
♂	8 years 9 months	European Shorthair	1
♂	10 years 7 months	Mix	1
♀	17 years 5 months	European Shorthair	1
♀	16 years 5 months	European Shorthair	1
♂	15 years 7 months	Mix	1
♂	6 years 1 month	Mix	1
♀	8 years 1 month	Mix	2
♂	N/A	European Shorthair	2
♀	15 years 5 months	European Shorthair	2
♂	16 years 11 months	European Shorthair	2
♀	3 years	Mix	2
♂	10 years 6 months	Mix	2
♂	11 years	European Shorthair	2
♀	15 years 1 month	European Shorthair	2
♂	11 years 8 months	European Shorthair	2
♂	16 years 8 months	European Shorthair	2
♀	15 years 1 month	European Shorthair	2
♀n	6 years 1 month	European Shorthair	2
♂	7 years	European Shorthair	2
♂	16 years 10 months	European Shorthair	2
♂	5 years 6 months	Mix	2
♂	13 years 7 months	European Shorthair	2
♂n	10 years 8 months	European Shorthair	2
♀n	8 years 8 months	European Shorthair	2
♀	5 years	European Shorthair	2
♂	7 years 1 month	European Shorthair	2
♀	15 years 11 months	European Shorthair	2
♀	7 years 1 month	European Shorthair	2

♂	7 years 1 month	European Shorthair	2
♀	11 years 1 month	European Shorthair	3
♂	9 years	European Shorthair	3
♂	4 years 9 months	Persian	3
♀n	13 years	European Shorthair	3
♀n	16 years 2 monthe	Burmese	3
♀	6 years 1 month	Mix	3
♂	13 years	Mix	3
♀	7 years 7 months	Siberian	3
♀	13 years 5 months	Persian	3
♂	10 years	European Shorthair	3
♂	N/A	European Shorthair	3
♀	N/A	European Shorthair	3
♀	14 years 11 months	European Shorthair	3
♂	12 years 5 months	Mix	3
♀	14 years	Mix	3
♀	9 years 2 months	Mix	3
♂n	6 years 5 months	European Shorthair	3
♂	8 years	European Shorthair	3
♀	4 years	Mix	3
♂	7 years 8 months	European Shorthair	3
♀	16 years 1 month	European Shorthair	3
♀	brak informacji	European Shorthair	4
♂	7 years 2 months	European Shorthair	4
♂	10 years 6 months	European Shorthair	4
♂	7 years 8 months	Mix	4
♂	10 years 1 month	European Shorthair	4
♀	9 years 11 months	European Shorthair	4
♂	6 years 9 months	European Shorthair	4
♂	8 years 1 month	Mix	4
♂n	8 years	European Shorthair	4
♂	5 years	European Shorthair	4
♀	6 years 2 months	European Shorthair	4
♂	2 years 5 months	European Shorthair	4
♂	8 years 4 months	European Shorthair	0
♂	3 years 10 months	European Shorthair	0
♂	10 years 3 months	Mix	0
♀	5 years 7 months	Mix	0
♀	4 years 11 months	European Shorthair	0
♂n	13 years 1 month	European Shorthair	0
♂n	11 years 4 month	European Shorthair	0
♀n	7 years 2 months	Persian	0
♀	7 years 9 months	European Shorthair	0

♀n – sterilized female; ♂n – sterilized male, 0 – healthy cats

Supplemetary table 2. Characteristic of dogs

Sex	Age	Breed	Clasification (group)
♂	13 years 1 month	Mix	1
♂	14 years 5 months	Mix	1
♂	15 years 5 months	West Highland white terrier	1
♂	7 years 8 months	Bulterier	1
♂	12 years 2 months	West Highland white terrier	1
♂	12 years	Mix	1
♂	10 years	Mix	1
♀	12 years	French Shepherd	1
♂	14 years 9 months	Mix	1
♀	14 years 1 month	Mix	1
♂	10 years 3 months	Maltese	1
♀	14 years 8 months	Shetland Sheepdog	1
♀	14 years 5 months	Yorkshire Terrier	1
♂	7 years	Polish Hunting Dog	1
♂	12 years 2 months	Mix	1
♂	4 years	N/A	1
♂	14 years 3 months	Pudelpointer	1
♂	14 years 1 month	Mix	1
♂	6 years 1 month	Mix	1
♂	15 years 7 months	Mix	1
♀	7 years	Labrador	1
♂	1 rok i 1month	Border Terrier	1
♀	19 years	Mix	1
♂	12 years 1 month	Mix	2
♂	15 years 1 month	Mix	2
♂	13 years 9 months	West Highland white terrier	2
♂	10 years 8 months	Labrador Retriever	2
♂	N/A	Mix	2
♀	12 years 5 months	Yorkshire Terrier	2
♀	17 years 4 months	Yorkshire Terrier	2
♂	9 years 10 months	Welsh Corgi Cardigan	2
♂	13 years 9 months	Husky	2
♀	4 years 9 months	French bulldog	2
♂	8 years 6 months	German Shepard	2
♂	14 years	Yorkshire Terrier	2
♂	5 years 6 months	Labrador Retriever	2
♂	12 years 2 months	Golden Retriever	2
♂	16 years	Mix	2
♀	13 years	Golden Retriever	2
♂	13 years	Mix	2
♂	14 years 3 months	Mix	2

♂	10 years 10 months	Labrador Retriever	2
♂	11 years 11 months	Mix	2
♀	11 years 5 months	Pekingese Palasthund	2
♀	8 years 11 months	Yorkshire Terrier	2
♀	16 years 4 months	Yorkshire Terrier	2
♂	12 years	Mix	2
♂	9 years	German Shepard	2
♀	10 years 7 months	Miniature Schnauzer	2
♂	17 years 5 months	Mix	2
♀	16 years 10 months	American Staffordshire Terrier	2
♀	13 years	Mix	3
♂	15 years	English Cocker Spaniel	3
♂	10 years	N/A	3
♀	3 years 4 months	Bernese Mountain Dog	3
♀	18 years 3 months	Mix	3
♀	14 years 4 months	Labrador Retriever	3
♀n	10 years 11 months	Mix	3
♂	14 years	West Highland white terrier	3
♂	6 years 6 months	Bearded Collie	3
♂	12 months	Wire-haired dachshund	3
♂	4 years 1 month	German Shepard	3
♀	14 years 5 months	Shih Tzu	3
♂	3 years 8 months	Border Collie	3
♀	10 years 1 month	Labrador Retriever	0
♀	8 years 11 months	Mix	0
♂	6 years 10 months	Mix	0
♂	10 years 1 month	Labrador Retriever	0
♂	3 years 3 months	Yorkshire Terrier	0
♂	16 years 3 months	Mix	0
♀n	14 years 1 months	Mix	0
♀n	3 years 7 months	West Highland white terrier	0
♂	12 years 2 months	Golden Retriever	0
♂	14 years 9 months	Labrador Retriever	0
♂n	6 years 1 month	Mix	0
♀	11 years 2 months	Mix	0
♀	5 years 8 months	Mix	0

♀n – sterilized female; ♂n – sterilized male, 0 – healthy dogs

Supplementary Table 3. Primer sequence used for Real Time PCR analysis.

Gene name	Forward (5’>3’)	Reverse (5’>3’)	Size (bp)
α-SMA dog	GTGGGGATGGGACAAAAGG	GAAAGCACCGCCTGAATAG	290
α-SMA cat	GCATGGGACAAAAGGACAG	TGGTGATGATGCCGTGTTC	59
TIMP1 dog	CAAGACCTATGCTGCTGGCT	CTGTGAGGAAGTGGTCCGTC	110
TIMP1 cat	CATCCTGTTGTTGCTGTGGC	TCTGGTTGACTTCTGCGGTC	134
Col1a dog	TCCTGGGCCTCAGGGTGCTC	GACCAGCAGGACCAGCATCTC	116
Col1a cat	CTGAAGGCTCTAGGAAGAAC	CATAGTGCATCCTTGGTTAG	62
FN1 dog	TGGAATGCCCCAGAACCATC	AAGCGTGTCACCTCTCTGTG	197

FN1 cat	CCCTCACCAATCTCACTCCG	CCCTCGGAACATCAGAAACTG	117
SPX dog	TGTCCAGGTGTCACGAAAGT	GCGCCCTTCAGATAGAGCAT	209
SPX cat	ATCTCGGACCAGAGTCGGAA	TGGTCTATTTCCCCAGGTTAGT	198
GALR1 dog	AGTCCGTAGAGCAGGTCCAT	CAGGGGAGCTCTTGAAGGTG	92
GALR1 cat	GAGGGTGTCGGATGTGTGTT	ATGCACCAGCCTGCTAATGT	148
GALR2 dog	CACCATCTACACCCTGGACGG	CGACTGGCGGTAGTAACTCA	253
GALR2 cat	CGGTTATCGTGCCCATGTTG	CAGCACACGATGAAGCACAG	160
GALR3 dog	CATGTATGCCAGCAGCTTCAC	GTGCCGTAGTAGCTGAGGTAG	174
GALR3 cat	GCCCTCGTTTGTAAGACGGT	GGTGCCGTAGTAGCTGAGAT	216
GAPDH dog	CTGGGGCTCACTTGAAAGG	CAAACATGGGGGCATCAG	22
GAPDH cat	GCCATCAATGACCCCTTCAT	GCCGTGGAATTTGCCGT	81

Supplementary figure 1. Correlation between SPX, Urea and creatinine in cats and dogs.

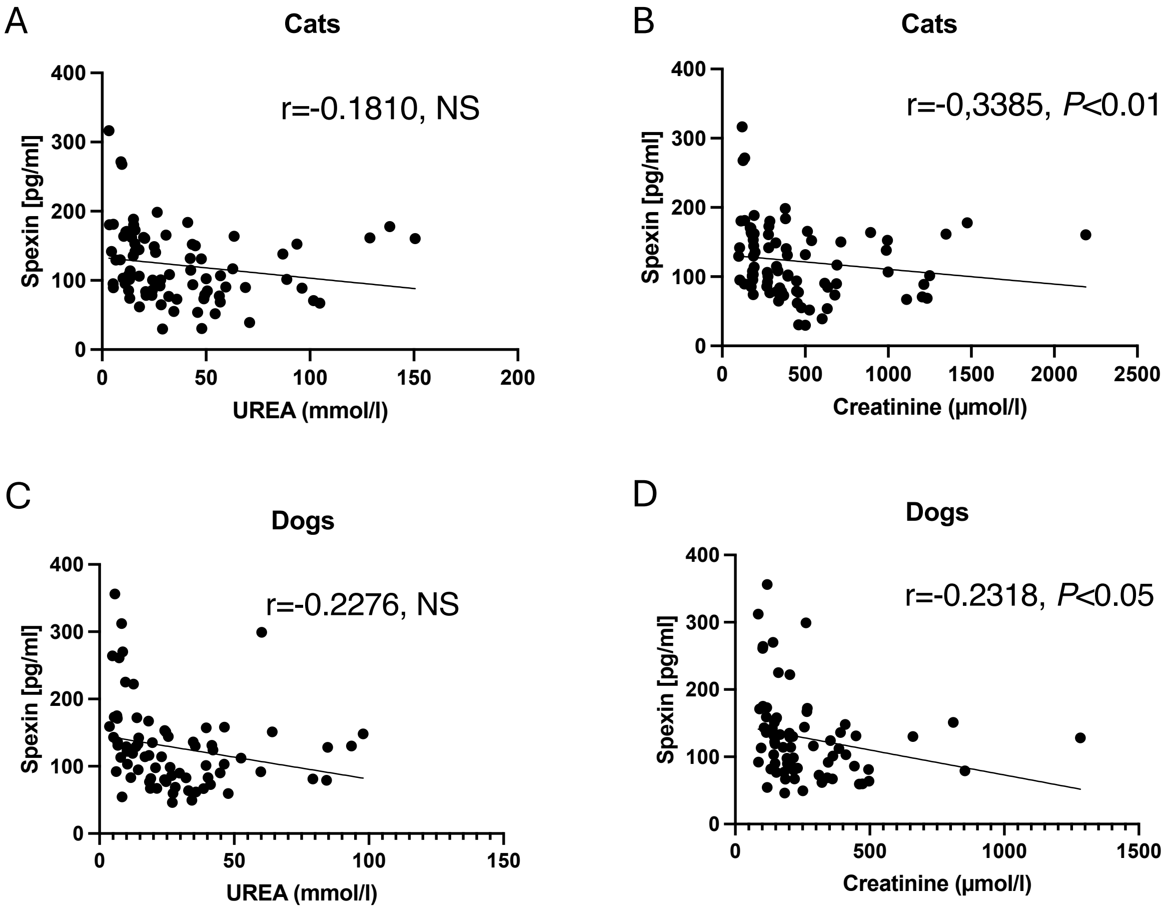

Correlation between urea, creatine and SPX in serum blood in cats (A and B) and dogs (C and D)
